# Prediction of Outcomes After Heart Transplantation in Pediatric Patients Using National Registry Data: Evaluation of Machine Learning Approaches

**DOI:** 10.2196/45352

**Published:** 2023-06-20

**Authors:** Michael O Killian, Shubo Tian, Aiwen Xing, Dana Hughes, Dipankar Gupta, Xiaoyu Wang, Zhe He

**Affiliations:** 1 College of Social Work Florida State University Tallahassee, FL United States; 2 Department of Statistics Florida State University Tallahassee, FL United States; 3 Congenital Heart Center, Shands Children’s Hospital University of Florida Gainesville, FL United States; 4 School of Information Florida State University Tallahassee, FL United States

**Keywords:** explainable artificial intelligence, machine learning, mortality, outcome prediction, organ rejection, organ transplantation, pediatrics, United Network for Organ Sharing

## Abstract

**Background:**

The prediction of posttransplant health outcomes for pediatric heart transplantation is critical for risk stratification and high-quality posttransplant care.

**Objective:**

The purpose of this study was to examine the use of machine learning (ML) models to predict rejection and mortality for pediatric heart transplant recipients.

**Methods:**

Various ML models were used to predict rejection and mortality at 1, 3, and 5 years after transplantation in pediatric heart transplant recipients using United Network for Organ Sharing data from 1987 to 2019. The variables used for predicting posttransplant outcomes included donor and recipient as well as medical and social factors. We evaluated 7 ML models—extreme gradient boosting (XGBoost), logistic regression, support vector machine, random forest (RF), stochastic gradient descent, multilayer perceptron, and adaptive boosting (AdaBoost)—as well as a deep learning model with 2 hidden layers with 100 neurons and a rectified linear unit (ReLU) activation function followed by batch normalization for each and a classification head with a softmax activation function. We used 10-fold cross-validation to evaluate model performance. Shapley additive explanations (SHAP) values were calculated to estimate the importance of each variable for prediction.

**Results:**

RF and AdaBoost models were the best-performing algorithms for different prediction windows across outcomes. RF outperformed other ML algorithms in predicting 5 of the 6 outcomes (area under the receiver operating characteristic curve [AUROC] 0.664 and 0.706 for 1-year and 3-year rejection, respectively, and AUROC 0.697, 0.758, and 0.763 for 1-year, 3-year, and 5-year mortality, respectively). AdaBoost achieved the best performance for prediction of 5-year rejection (AUROC 0.705).

**Conclusions:**

This study demonstrates the comparative utility of ML approaches for modeling posttransplant health outcomes using registry data. ML approaches can identify unique risk factors and their complex relationship with outcomes, thereby identifying patients considered to be at risk and informing the transplant community about the potential of these innovative approaches to improve pediatric care after heart transplantation. Future studies are required to translate the information derived from prediction models to optimize counseling, clinical care, and decision-making within pediatric organ transplant centers.

## Introduction

### Background

The rates of survival for pediatric solid organ transplant recipients continue to improve. Overall, the 5-year survival rate for pediatric heart transplant (HT) recipients was 81.5% between 2009 and 2013 [1]. Despite these improvements, ongoing concerns remain regarding the rates of late acute rejection (LAR) and hospitalization within this population [2-5]. Increased number and frequency of LAR episodes and hospitalizations reduce health-related quality of life of these patients and their families owing to multifactorial reasons [6-9]. Therefore, any insight to help stratify those patients at higher risk of posttransplant complications will allow better resource allocation and focused interventions to reduce morbidity and mortality.

With the advent of machine learning (ML) methodologies, predictive modeling has entered a new era, leveraging latent information from a large number of data points that was previously not practical. Despite advancements in research using ML and its predictive utility for prediction of posttransplant health outcomes, widespread use and clinical application are still limited in pediatric transplant recipients [10-12]. In addition, the currently available research into posttransplant health outcomes in pediatric patients has suffered from a lack of rigorous statistical approaches, small sample sizes comprising samples from single transplant centers with limited generalizability, and other methodological limitations [13-15]. Furthermore, general linear modeling or Cox proportional hazards regression approaches are prevalent in this research, offering limited predictive utility [16-18].

Data-driven modeling and ML approaches have had limited application in prediction of outcomes in pediatric heart transplantation despite the availability of robust databases of patient electronic health records (EHRs) and longitudinal data [19-21]. Among these few studies, the use of ML approaches in pediatric transplantation has resulted in limited success in predicting health outcomes [10,15,16]. However, the use of advanced ML approaches with these data are unexplored and can inform care and decision-making.

ML and deep learning (DL) approaches can identify unique risk factors as well as their complex relationship with outcomes using prediction modeling. Results from these approaches can thereby aid in identifying patients considered to be at high risk and provide a solid foundation for improved clinical care and risk stratification as well as enhance decision-making. In our previous work, DL and traditional ML techniques were applied to United Network for Organ Sharing (UNOS) patient data from a single large pediatric transplant center in the southwestern United States. Despite having to work with a relatively small sample, we demonstrated that traditional ML models can predict hospitalizations across liver, kidney, and heart transplantations with moderate accuracy [15]. This study sought to take a step further by testing and examining the utility of ML and DL models for predicting LAR and mortality at 1, 3, and 5 years after transplantation using national UNOS data on pediatric HT recipients. To the best of our knowledge, this is the first study that uses national registry data to evaluate ML-based prediction models for multiple post–heart transplantation outcomes across multiple prediction windows. In addition, the use of DL approaches with national UNOS data represents an important innovation for the prediction of posttransplant outcomes in pediatric patients. The long-term goal of this endeavor is to continue to improve the ability of pediatric transplant teams to identify patients early on who are at higher risk of poor posttransplant outcomes. Using the information gained from these modeling techniques will directly translate into the development of clinical decision-making support tools for pediatric transplantation teams and allow an opportunity to perform targeted interventions to potentially improve outcomes.

The remainder of this paper is organized as follows: in the *Related Work* subsection, we review the recent literature on building prediction models for outcomes of pediatric organ transplantation. In the *Methods* section, we describe the data set, problem setting, outcome definition, selection of variables, data preprocessing, ML and DL modeling, and model interpretation. In the *Results* section, we present the characteristics of the patient cohort, performance of the prediction models, and interpretation of the models. In the *Discussion* section, we discuss the principal findings, clinical meaningfulness of model interpretation, ways to improve modeling, and limitations, followed by a *Conclusions* subsection.

### Related Work

To identify related work in the literature, we searched PubMed for these terms in all text over the last 10 years: [(heart transplant*) AND (pediatric* or paediatric* or child* or adolescen*) AND (machine learning)]. A total of 123 studies were imported into Covidence (Veritas Health Innovation Ltd), a web-based software platform that facilitates conducting systematic reviews of research literature. Among the 123 studies, Covidence identified 22 (17.9%) duplicates. Next, we screened the remaining 101 studies using titles and abstracts and excluded 83 (82.2%) as irrelevant. Full-text review was conducted by multiple reviewers on the remaining 18 studies, of which 14 (78%) were ultimately excluded (n=7, 50%, did not use a pediatric sample or subsample; n=4, 29%, were not conducted using data from HT recipients; and n=3, 21%, did not use some form of ML or similar predictive modeling approach). Thus, of the initial 123 studies, 4 (3.3%; Table 1) were ultimately identified that predicted posttransplant health outcomes using ML with patient EHR data or administratively collected medical data of pediatric HT recipients. The literature search is documented in the PRISMA (Preferred Reporting Items for Systematic Reviews and Meta-Analyses) flowchart shown in Figure S1 in Multimedia Appendix 1.

**Table 1:** Related work in the literature.

Study	Prediction methods	Sample	Sample size, n	Outcomes	AUROC^a^, best (95% CI)	AUROC, best (outcome)
Gupta et al [11], 2022	Stepwise logistic regression, gradient boosting, and random forest	Pediatric Heart Transplant Society database; aged <18 years; heart transplantation; discernible discharge date; transplanted between January 2005 to December 2018	4414	Prolonged length of stay (>30 days) after transplantation	0.750 (0.720-0.780)	N/A^b^
Killian et al [15], 2021	Logistic regression, multilayer perceptron, sequential minimal optimization algorithm polynomial kernel, random forest, and deep learning	UNOS^c^ data for a single transplant center; aged 0-18 years; heart transplant; transplanted between 1988 and May 31, 2017	193	Hospitalization owing to rejection over 1-, 3-, and 5-year posttransplant periods	N/A	0.740 (5‐year hospitalization)
Miller et al [12], 2019	Artificial neural networks, classification and regression trees, and random forest	UNOS data; aged <18 years; heart transplant; transplanted between January 2006 and December 2016	2802	Mortality over 1-, 3-, and 5-year posttransplant periods	N/A	0.720 (1‐year mortality)
Miller et al [22], 2022	Random forest, XGBoost^d^, and L2 regularized logistic regression	UNOS data; aged <18 years; heart transplant; transplanted between January 1994 and December 2016	8349	1-year and 90-day all-cause mortality	0.836 (0.823-0.849)	N/A

^a^AUROC: area under the receiver operating characteristic curve.

^b^N/A: not applicable.

^c^UNOS: United Network for Organ Sharing.

^d^XGBoost: extreme gradient boosting.

Miller et al [12] conducted a study that involved pediatric patients from the UNOS database who underwent heart transplantation and aimed to predict mortality within 1, 3, or 5 years using artificial neural networks (NNs), classification and regression trees, and random forest (RF), and the area under the receiver operating characteristic curve (AUROC) values of the testing data were 0.72, 0.61, and 0.60, respectively. All models displayed poor sensitivity in identifying positive cases, and the authors explained that the ML algorithm tended to be biased toward the common outcomes rather than toward the rarities. In a more recent study, Miller et al [22] used 3 binary classification algorithms (RF, extreme gradient boosting [XGBoost], and L2 regularized logistic regression [LR]) and 3 survival models (random survival forest, survival gradient boosting, and L2 regularized Cox regression) to predict 1-year and 90-day mortality after heart transplantation. The study used shuffled 10-fold cross-validation (CV) and rolling CV where each fold is a transplantation year, and training data are from at least 1 transplantation year before the evaluated year. In the shuffled CV, RF was the best-performing model, and it achieved a much better performance (AUROC 0.893, 95% CI 0.889-0.897) than XGBoost, which was the best model in the rolling CV (AUROC 0.657, 95% CI 0.647-0.667), indicating that the overprediction performance is limited by the temporal shift in the data. Our study differs from the work by Miller et al [22] in that we compared the performance of mortality and organ rejection prediction models. We also used Shapley additive explanations (SHAP), a post hoc explanation method, to rank the features by their importance.

Gupta et al [11] analyzed the data in the Pediatric Heart Transplant Society database and identified factors that are related to the prolonged length of stay (>30 days) after heart transplantation among pediatric patients. This study evaluated stepwise LR, gradient boosting, and RF when building the risk-prediction model for prolonged length of stay. The final prediction model achieved an AUROC value of 0.75 (95% CI 0.72-0.78) for the overall population. Killian et al [15] extracted the data of pediatric patients who underwent heart, kidney, or liver transplantation from UNOS data from a single transplant center in the United States and focused on the prediction of hospitalization within the observation windows of 1, 3, and 5 years after each patient’s first organ transplantation using both traditional ML methods (RF, LR, multilayer perceptron [MLP], and support vector machine [SVM]) and a simple feed-forward NN model. The overall performance of DL was not better than that of the traditional ML methods. The best-performing model was the RF model for 5-year hospitalization prediction (AUROC 0.74). Our study differs from the work by Killian et al [15] in three aspects: (1) we used national UNOS data for the modeling, (2) we built models to predict organ rejection and mortality outcomes and compared them, and (3) we used the observation data collected up to the time of the transplantation procedure to predict the outcomes.

## Methods

### UNOS Data

For this study, we used national UNOS data from 1987 to 2019 [23]. This database contains pretransplant medical information and long-term and posttransplant health outcomes of organ transplant recipients at the national and center level. A record of each recipient in the UNOS data is established when the recipient is registered as a candidate for an organ transplant. Each recipient’s record includes their pre- and posttransplant medical and health data completed at 3 time points: being listed for a transplant (ie, transplant candidate registration), at the time of the transplant procedure (ie, transplant recipient registration), and annually as a posttransplant follow-up (ie, transplant recipient follow-up [TRF]). Information related to pretransplant conditions, medical data concerning the transplant procedure, posttransplant complications, and long-term health outcomes are also collected and reported by the transplant centers. These data were stored in the corresponding variables, which were then used as predictors and responses for different ML and DL models.

**Figure 1:**
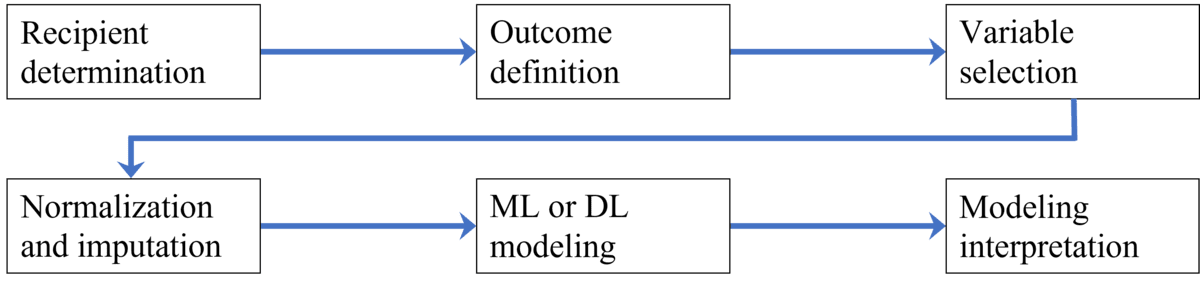
Overall workflow. DL: deep learning; ML: machine learning.

The overall workflow for this study is shown in Figure 1. After the identification of the patient cohort, we defined the prediction outcomes and chose the observation and prediction windows. Relevant variables were selected based on previous studies [17,24-31] and chosen by a medical expert from the available data as potential predictors. Subsequently, data normalization and imputation were performed, followed by ML and DL modeling and modeling interpretation. Details of each step are explained in the following subsections.

### Recipient Determination

The target recipients for this study are primary pediatric HT recipients aged 0 to 18 years. The exclusion criteria were as follows: retransplantation, records with missing follow-up dates, no follow-up information during the prediction window, and patients with unknown or missing values in their outcome variables. Table 2 shows the basic demographic characteristics of the entire cohort.

**Table 2:** Characteristics of the entire patient cohort.^a^

Recipient			Overall (N=8201)	Alive or unknown (n=5887)		Deceased (n=2314)		*P* value	
Age (years), mean (SD)			6.78 (6.48)	6.39 (6.38)		7.76 (6.62)		<.001	
Sex (female), n (%)			3577 (43.62)	2558 (43.45)		1019 (44.04)		.63	
**Race, n (%)**									<.001
	American Indian or Alaska Native	41 (0.5)		25 (0.42)	16 (0.69)				
	Asian	287 (3.5)		235 (3.99)	52 (2.25)				
	Black or African American	1591 (19.4)		970 (16.48)	621 (26.84)				
	Native Hawaiian or other Pacific Islander	29 (0.35)		13 (0.22)	16 (0.69)				
	White	4781 (58.3)		3505 (59.54)	1276 (55.14)				
	Multiracial	150 (1.83)		113 (1.92)	37 (1.6)				
Ethnicity (Hispanic), n (%)			1317 (16.06)	1023 (17.38)		294 (12.71)		<.001	
BMI (kg/m^2^), mean (SD)			17.64 (4.92)	17.53 (4.8)		17.91 (5.2)		.002	
Height (cm), mean (SD)			110.28 (44.42)	107.70 (43.88)		116.80 (45.12)		<.001	
**Education, n (%)**									<.001
	None	102 (1.4)		76 (1.37)	28 (1.46)				
	Grade school (grades 0-8)	2044 (28)		1551 (27.87)	543 (28.24)				
	High school (grades 9-12) or GED^b^	1149 (15.74)		809 (14.54)	370 (19.24)				
	Attended college or technical school	32 (0.44)		22 (0.4)	14 (0.73)				
	Associate or bachelor’s degree	1 (0.01)		1 (0.02)	0 (0)				
	N/A^c^ (aged <5 years old)	3886 (51.9)		2986 (53.66)	900 (46.8)				
Prior cardiac surgery, n (%)			404 (8.79)	270 (9.95)		134 (7.12)		<.001	
**Diabetes, n (%)**									.13
	No	7021 (97.7)		5284 (97.91)	1737 (97.09)				
	Type 1	16 (0.22)		12 (0.22)	4 (0.22)				
	Type 2	8 (0.11)		7 (0.13)	1 (0.06)				
Serum creatinine (mg/dL), mean (SD)			0.64 (1.23)	0.6 (1.08)		0.77 (1.6)		<.001	
CMV^d^+, positive, n (%)			2286 (30.63)	1768 (32.39)		518 (25.84)		<.001	
EBV^e^+, positive, n (%)			2977 (50.03)	2321 (49.37)		656 (52.48)		<.001	
**ABO^f^ match, n (%)**									<.001
	Identical	6353 (77.47)		4536 (77.05)	1817 (78.52)				
	Compatible	1616 (19.7)		1154 (19.6)	462 (19.97)				
	Incompatible	232 (2.83)		197 (3.35)	35 (1.51)				
**Primary diagnosis, n (%)**									
	Cardiomyopathy	4272 (52.09)		3092 (52.52)	1180 (50.99)		.21		
	CHD^g^	3638 (44.36)		2590 (44)	1048 (45.29)		.29		
	Other	291 (3.55)		205 (3.48)	86 (3.72)		.61		
**Secondary diagnosis, n (%)**									
	CHD with HLHS^h^	85 (1.04)		65 (1.10)	20 (0.86)		.33		
	CHD with prior surgery	1700 (20.73)		1388 (23.58)	312 (13.48)		<.001		
	Dilated myopathy	3588 (43.75)		2554 (43.38)	1034 (44.68)		.29		
	Hypertrophic cardiomyopathy	228 (2.78)		175 (2.97)	53 (2.29)		.09		
	Restrictive myopathy	442 (5.39)		349 (5.93)	93 (4.02)		.001		
**Ventricular assist device, n (%)**									<.001
	None	4180 (78.19)		3401 (78.45)	779 (77.05)				
	LVAD^i^	761 (14.23)		650 (14.99)	111 (10.98)				
	RVAD^j^	16 (0.3)		12 (0.28)	4 (0.4)				
	TAH^k^	6 (0.11)		6 (0.14)	0 (0)				
	LVAD + RVAD	214 (4)		178 (4.11)	36 (3.56)				
	LVAD, RVAD, or TAH unspecified	169 (3.16)		88 (2.03)	81 (8.01)				
**Year of transplant (range), n (%)**									<.001
	1987-1990	387 (4.73)		202 (3.43)	185 (8)				
	1991-1995	1074 (13.1)		523 (8.88)	551 (23.82)				
	1996-2000	1150 (14.03)		658 (11.18)	492 (21.26)				
	2001-2005	1255 (15.3)		807 (13.72)	448 (19.37)				
	2006-2010	1548 (18.88)		1185 (20.13)	363 (15.68)				
	2011-2015	1877 (22.88)		1652 (28.05)	225 (9.73)				
	2016-2018	910 (11.09)		860 (14.61)	50 (2.16)				
Days listed, mean (SD)			95.33 (196.89)	99.43 (209.3)		84.92 (160.5)		.003	
Days listed as status 1A^l^, mean (SD)			32.92 (61.31)	37.71 (61.94)		20.73 (57.93)		<.001	

^a^Nonmissing values are used to calculate summary statistics, frequency, and percentages.

^b^GED: General Educational Development Test.

^c^N/A: not applicable.

^d^CMV: cytomegalovirus.

^e^EBV: Epstein-Barr virus.

^f^ABO: the 4 main blood types are A, B, O, and AB; for a blood transfusion, the ABO blood group system is used to match the blood type of the donor and the person receiving the transfusion.

^g^CHD: congenital heart defect.

^h^HLHS: hypoplastic left heart syndrome.

^i^LVAD: left ventricular assist device.

^j^RVAD: right ventricular assist device.

^k^TAH: total artificial heart.

^l^Status 1A: the United Network for Organ Sharing status code 1A is the most severe designation for need for transplantation. Candidates on the waiting list at this level are critically ill and are receiving some form of mechanical circulatory support.

### Outcome Definition

**Figure 2:**
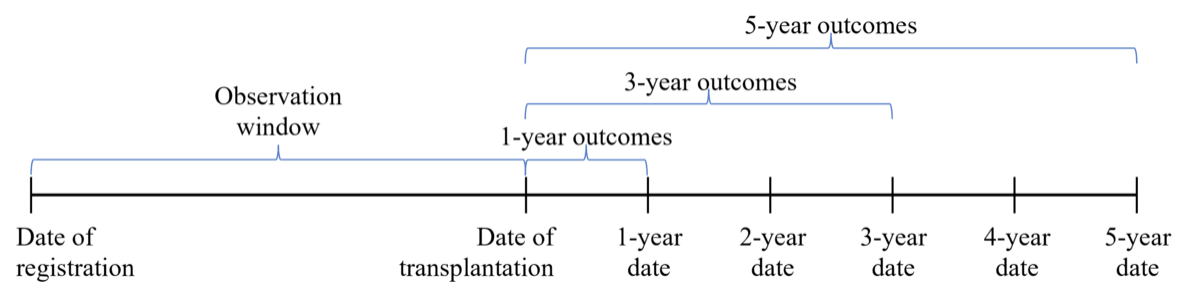
Observation window and outcome prediction windows.

In this study, we studied 2 prediction outcomes: rejection and mortality after transplantation. For each prediction outcome (rejection or mortality), we considered 3 different outcome prediction windows of 1, 3, and 5 years after transplantation. The observation window used was the information from baseline data collected at listing or registration for a transplant and immediately after the transplant procedure. The data collected from the observational window were used as the predictors. For the prediction window of 1-year outcomes, we used the last TRF information of each patient within 1 year after transplantation to determine the 1-year outcomes. Similarly, for the prediction window of 3-year outcomes, outcomes were determined using the annual follow-up information of each patient from the time of transplantation until 3 years after transplantation. For the prediction window of 5-year outcomes, outcomes were determined using the annual follow-up information of each patient from the time of transplantation until 5 years after transplantation. Figure 2 illustrates the observation window and the outcome prediction windows for this study.

In the UNOS data, rejection outcome was defined by 2 variables jointly: *hospitalized for rejection during follow-up period* (HOSP_REJ) and *episodes of acute rejection* (ACUTE_REJ_EPI). In the study period, UNOS used these variables at different times: HOSP_REJ from April 1, 1994, and ACUTE_REJ_EPI from June 30, 2004. Therefore, we used these variables as such to define presence or absence of rejection. Therefore, rejection was determined with HOSP_REJ before June 30, 2004; after June 30, 2004, the rejection outcome was positive if either HOSP_REJ or ACUTE_REJ_EPI was *Yes* and negative otherwise. Mortality was determined using the corresponding variables from the UNOS thoracic follow-up data set. The latest collection date for pediatric HT recipients was February 28, 2019, in the data set. Tables 3 and 4 show the number of valid recipients with known prediction outcome in each prediction window. Table 5 shows the number of patients included in data sets for predicting outcomes in multiple prediction windows.

**Table 3:** Number of valid recipients with known rejection prediction outcome in each prediction window.

Rejection	1-year prediction window (n=2882), n (%)	3-year prediction window (n=2582), n (%)	5-year prediction window (n=2709), n (%)
No	2100 (72.87)	553 (21.42)	225 (8.31)
Yes	782 (27.13)	2029 (78.58)	2484 (91.69)

**Table 4:** Number of valid recipients with known mortality prediction outcome in each prediction window.

Mortality	1-year prediction window (n=6035), n (%)	3-year prediction window (n=3306), n (%)	5-year prediction window (n=2237), n (%)
No	5608 (92.92)	2388 (72.23)	969 (43.32)
Yes	427 (7.08)	918 (27.77)	1268 (56.68)

**Table 5:** Patients appearing in data sets for different prediction windows.

Characteristics	Outcomes	
	Rejection, n	Hospitalization, n
Have outcomes in year 1 and year 2 or 3 but not in year 4 or 5	47	116
Have outcomes in year 1 and year 4 or 5 but not in year 2 or 3	10	35
Have outcomes in year 2 or 3 and year 4 or 5 but not in year 1	61	174
Have outcomes in year 1, year 2 or 3, and year 4 or 5	66	277

### Selection of Variables

Through literature review, we selected common features in UNOS data in prediction models for transplantation outcome predictions [17,24-31]. The variables were selected from donor, recipient, and donor-recipient variables. In addition, a medical expert and coauthor (DG) reviewed the list of identified features and determined the ones that were clinically relevant and should be used in predictive modeling. In addition, *diagnosis* was selected as a variable and included congenital heart defect (CHD), CHD with hypoplastic left heart syndrome, cardiomyopathy, CHD with prior surgery, dilated cardiomyopathy, hypertrophic cardiomyopathy, restrictive myopathy, and other. Any variables with >50% missing values were excluded from analysis.

### Normalization and Imputation

The selected variables included categorical and continuous numerical variables. Categorical variables were coded into numerical variables for computation. The values of all continuous numerical variables were normalized between 0 and 1. Because of missing values, we conducted a missing data imputation using multivariate imputation by chained equations [32]. After normalization and imputation, variables that were collinear with other variables were excluded. This process resulted in a list of the 69 selected variables in different groups. Description and type of each variable are provided in Table S1 in Multimedia Appendix 1. Details of coding for each categorical variable can be found in Table S2 in Multimedia Appendix 1.

### ML and DL Modeling

In this study, 7 ML models and 1 DL model were tested. The ML models were XGBoost, LR, SVM, RF, stochastic gradient descent, MLP, and adaptive boosting (AdaBoost). We used the *scikit-learn* package in Python (Python Software Foundation) for the implementation of all ML models. All ML models were implemented with default settings. The DL model was implemented with the Python packages of *TensorFlow* and *Keras*. After experimenting with different hyperparameters, the selected DL model included 2 hidden layers with 100 neurons and a rectified linear unit (ReLU) activation function followed by batch normalization for each and a classification head with a softmax activation function. The model used the adaptive gradient algorithm with a learning rate of 0.01 as optimizer and used cross-entropy as loss function. We trained the DL model for 50 epochs at most, with batch size of 32 and early stopping. The evaluation metrics reported include weighted precision, weighted recall, weighted *F*_1_-score, weighted AUROC values, and area under the precision-recall curve (AUPRC) values. AUROC measures the model’s ability to distinguish between positive and negative classes, whereas AUPRC measures the trade-off between precision and recall. AUPRC is often considered when the data sets used to build the models are imbalanced. We used 10-fold CV to evaluate all ML models. In each fold, a random sample of 90% of the instances were used for training, and the remaining 10% of the samples were used for testing. All evaluation metrics were computed using 10-fold CV for all models. The performances of the tested ML and DL models are reported in the Results section.

### Modeling Interpretation

Prediction results of ML and DL models are often considered difficult, and sometimes even impossible, to interpret for both users and developers. With the widespread application of ML and DL, understanding why a model makes a certain prediction becomes even more important. This has led to many research studies in the field of explainable artificial intelligence [33]. These studies have proposed, developed, and tested a wide range of methods for interpreting prediction results of ML and DL models. Among these methods, SHAP provides a state-of-the-art unified framework for explainable artificial intelligence.

SHAP is an additive feature attribution approach for interpreting prediction results of an ML or DL model [34]. It assigns an importance value to each feature for a particular prediction using the classic Shapley values from game theory and their related extensions. SHAP values are attributed to the change in the expected model prediction compared with the base model fitted on background data when conditioning on each feature. The implementation of SHAP is publicly available on GitHub [35]. In this study, we used SHAP to interpret prediction results of the best-performing ML model: RF. We used the SHAP *TreeExplainer* for the interpretation of RF predictions in terms of predicted probabilities. Details of interpretation are explained in the *Results* section.

### Ethical Considerations

In this study, we used publicly available deidentified UNOS data. Therefore, it was determined as exempt by the institutional review board of Florida State University.

## Results

### Characteristics of the Patient Cohort

Our cohort consisted of 8201 patients (UNOS data from 1987 to 2019), of whom 5887 (71.78%) were alive at the time of analysis. The characteristics of the overall patient cohort are shown in Table 2. Overall, the mean age of the cohort was 6.78 (SD 6.48) years, and 43.62% (3577/8201) of the patients were female. Interestingly, important differences were observed in race distribution, prior cardiac surgeries, and frequency of renal dysfunction between the patients who were deceased and those who were alive. There were significantly more Black or African American patients in the deceased group than the alive group (621/2314, 26.84% vs 970/5887, 16.48%; *P*<.001). No statistically significant difference was observed with a primary diagnosis of CHD (*P*=.29) or cardiomyopathy (*P*=.21) as the reason for transplantation. Furthermore, the diagnosis of CHD with prior surgeries (*P*<.001), prior cardiac surgery (*P*<.001), and restrictive cardiomyopathy (*P*=.005) was seen more frequently in the alive group. However, the number of valid recipients for each prediction window of the 2 different outcomes varied (Tables 3 and 4); for example, there were 2882 recipients with regard to the question on rejection within 1 year, of whom 2100 (72.87%) had no episodes of rejection, whereas 782 (27.13%) had episodes of rejection. Overall, the frequency distributions of episodes of rejection at 1, 3, and 5 years after transplantation were 27.13% (782/2882), 78.58% (2029/2582), and 91.69% (2484/2709), respectively (Table 3). Similarly, the frequency distributions of 1-, 3- and 5-year mortality outcomes were 7.08% (427/6035), 27.77% (918/3306), and 56.68% (1268/2237), respectively (Table 4).

### Performance of the Predictive Models

The performance details of each of the tested models are reported in Table 6. We observed that there was a variation in the type of model performance with some of the models performing better than others for some outcomes. When considering AUROC as the key performance evaluation measure, RF outperformed other ML and DL algorithms in predicting 5 of the 6 outcomes (all except 5-year rejection; AUROC 0.664 and 0.706 for 1-year and 3-year rejection, respectively, and AUROC 0.697, 0.758, and 0.763 for 1-year, 3-year, and 5-year mortality, respectively). For the 5-year rejection prediction, the AdaBoost model achieved the best performance (AUROC 0.705).

**Table 6:** Performance of different prediction models for rejection and mortality.

Prediction models				Precision	Recall		*F*_1_-score		AUROC^a^	AUPRC^b^
**Rejection**										
	**At 1 year**									
		XGBoost^c^	0.688		0.726	0.691		0.641		0.576
		LR^d^	0.698		0.737	0.679		0.648		0.576
		SVM^e^	0.531		0.728	0.614		0.485		0.614
		RF^f^	0.695		0.735	0.677		0.664		0.575
		SGD^g^	0.641		0.611	0.623		0.547		0.592
		MLP^h^	0.662		0.712	0.668		0.627		0.578
		AdaBoost^i^	0.699		0.735	0.696		0.648		0.576
		NN^j^	0.610		0.699	0.629		0.504		0.604
	**At 3 years**									
		XGBoost	0.717		0.768	0.728		0.695		0.739
		LR	0.709		0.779	0.711		0.692		0.737
		SVM	0.617		0.785	0.691		0.480		0.663
		RF	0.724		0.785	0.707		0.706		0.738
		SGD	0.680		0.677	0.679		0.523		0.668
		MLP	0.697		0.766	0.712		0.675		0.733
		AdaBoost	0.717		0.769	0.728		0.703		0.734
		NN	0.673		0.780	0.694		0.491		0.664
	**At 5 years**									
		XGBoost	0.873		0.915	0.881		0.697		0.888
		LR	0.841		0.916	0.877		0.685		0.885
		SVM	0.841		0.917	0.877		0.462		0.841
		RF	0.841		0.917	0.877		0.676		0.882
		SGD	0.853		0.816	0.833		0.526		0.851
		MLP	0.847		0.905	0.873		0.667		0.882
		AdaBoost	0.866		0.911	0.880		0.705		0.887
		NN	0.853		0.915	0.877		0.484		0.847
**Mortality**										
	**At 1 year**									
		XGBoost	0.878		0.926	0.896		0.663		0.838
		LR	0.899		0.929	0.895		0.669		0.835
		SVM	0.863		0.929	0.895		0.502		0.868
		RF	0.863		0.929	0.895		0.697		0.834
		SGD	0.875		0.912	0.891		0.534		0.859
		MLP	0.887		0.928	0.897		0.652		0.837
		AdaBoost	0.886		0.926	0.898		0.667		0.838
		NN	0.863		0.927	0.894		0.493		0.868
	**At 3 years**									
		XGBoost	0.725		0.745	0.729		0.737		0.567
		LR	0.709		0.739	0.699		0.719		0.566
		SVM	0.626		0.722	0.607		0.574		0.584
		RF	0.718		0.745	0.706		0.758		0.569
		SGD	0.646		0.596	0.614		0.564		0.584
		MLP	0.707		0.735	0.707		0.711		0.567
		AdaBoost	0.720		0.744	0.720		0.738		0.565
		NN	0.603		0.677	0.623		0.503		0.600
	**At 5 years**									
		XGBoost	0.688		0.690	0.689		0.748		0.575
		LR	0.668		0.671	0.669		0.718		0.559
		SVM	0.577		0.588	0.555		0.613		0.530
		RF	0.717		0.718	0.717		0.763		0.574
		SGD	0.599		0.604	0.600		0.596		0.521
		MLP	0.636		0.638	0.622		0.683		0.550
		AdaBoost	0.692		0.692	0.692		0.735		0.562
		NN	0.508		0.534	0.501		0.517		0.514

^a^AUROC: area under the receiver operating characteristic curve.

^b^AUPRC: area under the precision-recall curve.

^c^XGBoost: extreme gradient boosting.

^d^LR: logistic regression.

^e^SVM: support vector machine.

^f^RF: random forest.

^g^SGD: stochastic gradient descent.

^h^MLP: multilayer perceptron.

^i^AdaBoost: adaptive boosting.

^j^NN: neural network.

When examining the performance of the tested models across different prediction outcomes, the AUROC values for models predicting mortality were considerably higher than those of models predicting rejection (mean AUROC for rejection prediction 0.610, SD 0.090, and mean AUROC for mortality prediction 0.648, SD 0.091; *P*<.001).

When comparing the performance of the tested models across different prediction windows of each outcome, there is no significant difference among the AUROC values of the models for different prediction windows of rejection at significance level of .01. However, the AUROC value of the models for the 1-year prediction window of mortality is lower than the AUROC values of the models for the 3-year and 5-year prediction windows of mortality.

With respect to AUPRC values, XGBoost outperformed the other models in 3 of the 6 outcomes (ie, AUPRC 0.739 for 3-year rejection, AUPRC 0.888 for 5-year rejection, and AUPRC 0.575 for 5-year mortality). The NN outperformed other models in 2 outcomes (ie, AUPRC 0.868 for 1-year mortality and AUPRC 0.600 for 3-year mortality). For the 1-year rejection prediction, the SVM performed slightly better than the NN (AUPRC 0.614). Among all outcomes, the prediction of 1-year mortality and 5-year rejection showed significantly better performance than the prediction of other outcomes (mean AUPRC for 1-year mortality prediction 0.847, SD 0.015, and mean AUPRC for 5-year rejection prediction 0.870, SD 0.020).

**Figure 3:**
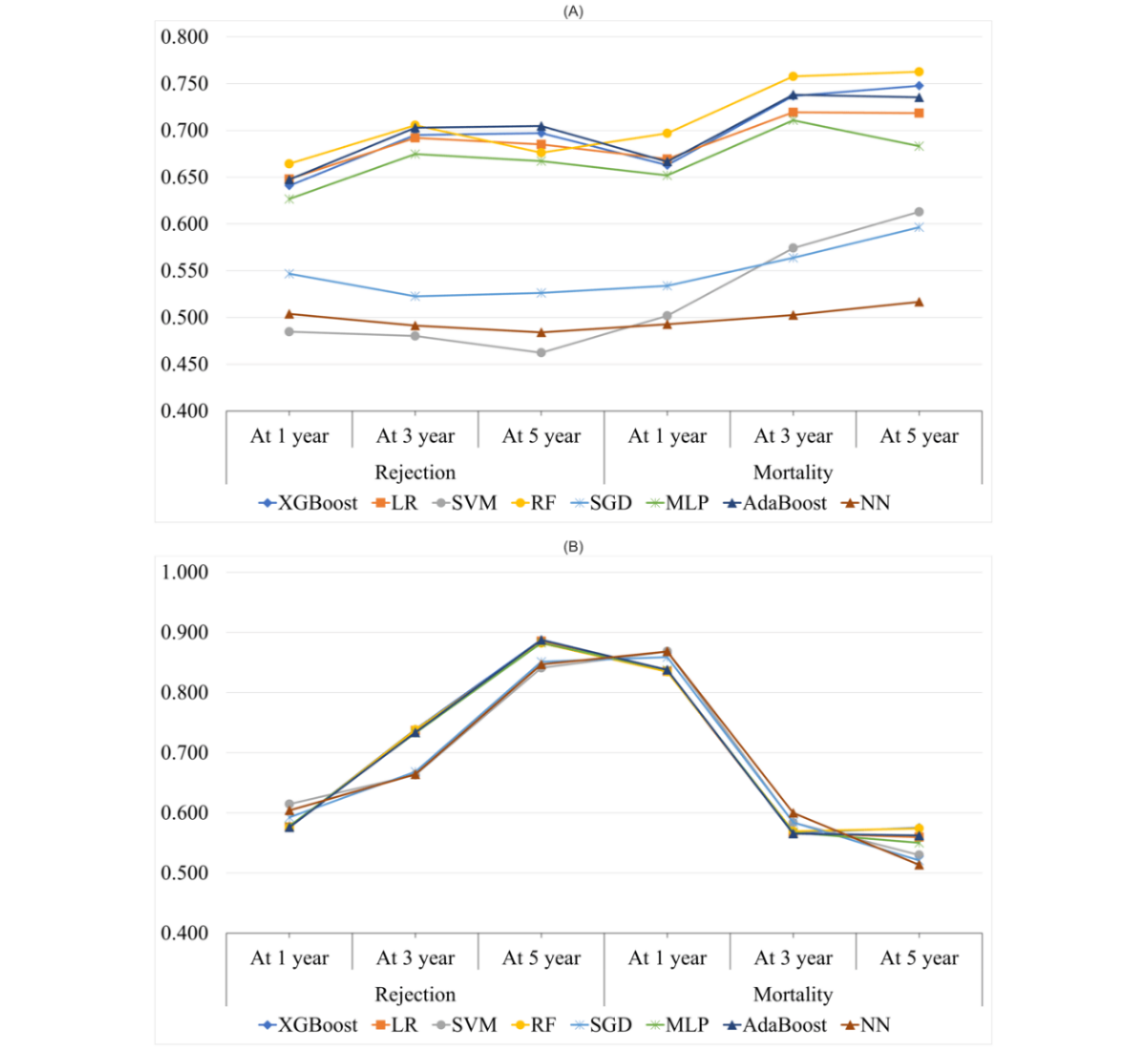
(A) Area under the receiver operating characteristic curve values of different machine learning and deep learning algorithms for different outcomes. (B) Area under the precision-recall curve values of different machine learning and deep learning algorithms for different outcomes. AdaBoost: adaptive boosting; LR: logistic regression; MLP: multilayer perceptron; NN: neural network; RF: random forest; SGD: stochastic gradient descent; SVM: support vector machine; XGBoost: extreme gradient boosting.

In Figure 3, we show a comparison of the performances of different models across different prediction windows and outcomes. When we evaluated the AUROC values of different algorithms across different prediction windows and outcomes, we observed that the DL model consistently had worse performance than the other algorithms. This finding is also consistent with our previous analysis, which used data from a single transplant center in the southwestern United States [15].

### Interpretation of the Best-Performing Models by SHAP Value

**Figure 4:**
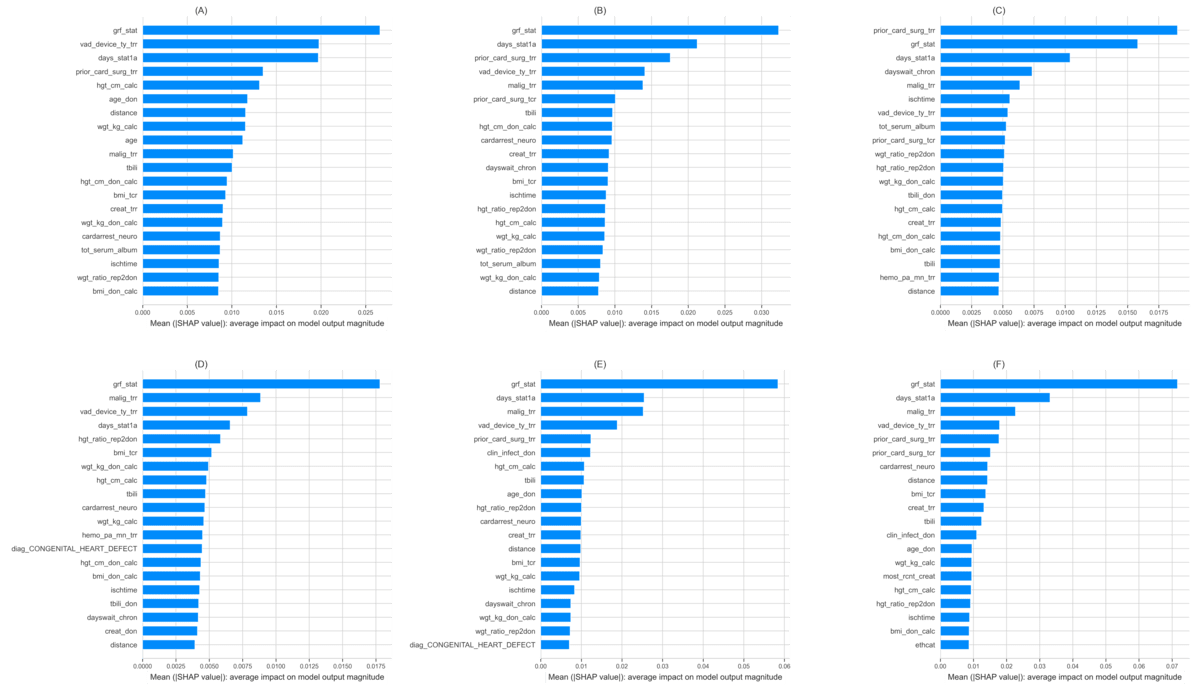
Impact of the top 20 variables on rejection and mortality prediction by mean (|SHAP value|) for the random forest model. (A) Rejection: 1-year window. (B) Rejection: 3-year window. (C) Rejection: 5-year window. (D) Mortality: 1-year window. (E) Mortality: 3-year window. (F) Mortality: 5-year window. SHAP: Shapley additive explanations. For a higher-resolution version of this figure, see Multimedia Appendix 2.

Figure 4 demonstrates the impact of 20 predictor variables in terms of mean (|SHAP value|) on the outcome prediction results of RF models. The length of each bar indicates the strength of the impact the corresponding variable has on the model prediction. An examination of the impact of the predictor variables in terms of mean (|SHAP value|) across all RF models suggests that, overall, the recipient variables of graft status after transplantation, education, any known malignancies since listing for transplantation, ethnicity, and height, as well as donor height and weight, have a higher impact on prediction. In addition, graft status immediately after the transplantation was a salient predictor in nearly every model and often the most predictive per SHAP value. Pretransplant medical factors such as prior cardiac surgeries, the diagnosis of a congenital heart condition, and the use of ventricular assist devices and mechanical ventilation before the transplant procedure were important predictors across models and outcomes. Patient medical factors that were shown to be predictive included weight; a history of prior malignancies; and albumin, bilirubin, and creatinine levels. Furthermore, factors such as donor cause of death, ischemic time, waitlist duration, and duration of time listed as status 1A (the UNOS status code 1A is designated for candidates on the waiting list who have the highest priority on the basis of medical urgency; patients may be listed as status 1A for 30 days at any time after left ventricular assist device implantation when they are clinically stable) were found to be predictive.

Table S3 in Multimedia Appendix 1 shows the predictor variables that have higher impact on prediction by outcome, prediction window, and ML algorithm according to SHAP value.

## Discussion

### Principal Findings

In this study, we compared 7 ML models and 1 DL model and examined their ability to predict rejection and mortality 1, 3, and 5 years after pediatric heart transplantation. There has been increasing use of advanced mathematical modeling using large data sets to predict outcomes in pediatric transplantation [10-12]. However, despite initial experience, much work needs to be done to further evaluate and refine the best strategies and modeling techniques to optimally use these methods for advancing clinical care. In this study, RF, XGBoost, and AdaBoost demonstrated the highest AUROC values throughout the posttransplant outcomes across the 3 observation windows. As a decision tree–based ensemble ML algorithm, RF has been shown to yield the best performance in many other studies on small, tabulated data sets, which is also the case in our study. A possible reason is that RF generally performs well when the data set has a mix of categorical and numeric features; in addition, RF is less influenced by outliers than other algorithms. Nonetheless, based on best practice in ML modeling, one would need to experiment with multiple ML algorithms on a particular data set to see which ML model works best. In our study, when AUPRC was used as the primary performance measure, XGBoost outperformed other models in 3 of the 6 outcomes and yielded slightly better performance than RF. The NN slightly outperformed other models in 2 outcomes. Most importantly, the use of SHAP values to evaluate the relative importance of predictors in these models adds to the clinical interpretability, utility, and potential translation into clinical care. We also observed that the DL model consistently had worse performance than the ML algorithms, which may be related to the small amount of data available because, empirically, DL models perform better with a large number of data points. This can also suggest that DL modeling in this clinical scenario may not be the most appropriate strategy. This finding is also consistent with our previous analysis, which used data from a single transplant center in the southwestern United States [15]. However, further research is needed to validate this conclusion.

The results from this modeling demonstrate the important challenges of using registry and administrative data to model adverse medical events during posttransplant care of pediatric HT recipients. Prior research and modeling of posttransplant data in pediatric care similarly found poor-to-fair predictive utility and sensitivity using classification and regression trees, RF, and artificial NN approaches [10-12]. Previous research using RF has identified key factors in predicting ideal posttransplant outcomes 3 years after liver transplantation [10]. However, results from ML models in pediatric transplantation across kidney, liver, and heart recipients from 1 center were similarly suboptimal [15]. In adult populations, predictive validity with ML approaches has not achieved encouraging results [28,36-43]. Many of these studies have focused only on mortality in adult HT recipients, offering little insight for pediatric transplant teams managing instances of other important outcomes such as rejection in a much more heterogenous population. Despite the UNOS being the largest registry of data for pediatric transplant patients, there are inherent data quality issues that may limit the optimal use of these analytical approaches. Therefore, urgent efforts are needed to improve quality of data entry and reduce the amount of missing data.

### Model Interpretation

SHAP values [34] were used in this study to provide greater interpretability of the results and to quantify the relative influence of individual variables within these models. Our data highlight the importance of graft status immediately after transplantation as being a salient predictor in nearly every model. Graft function immediately after transplantation is affected by a complex interplay of donor, preservation, recipient, and perioperative factors. These factors are unique in individual patients; however, the presence of suboptimal graft function immediately after transplantation is a strong predictor of 1-, 3-, and 5-year rejection and mortality. This observation does not necessarily change clinical management currently; however, it highlights the importance of in-depth evaluation and optimization of donor, recipient, and transplantation factors, which can influence graft function and the strength of its influence on important clinical outcomes; for example, donor myocardial function, ischemic time, and sensitization are a few factors that can influence graft function after transplantation. Other factors such as pretransplant use of ventricular assist devices and mechanical ventilation are important factors in predicting clinical outcomes as well. Furthermore, liver or kidney dysfunction and being listed as status 1A, all of which can be considered surrogate markers for a patient who is sicker, have important predictive influence on the outcomes. Various donor factors such as weight, height, and BMI, as well as recipient-to-donor weight ratio, influenced the predictive models. We hypothesize that these factors were likely related to the smaller children who are more likely to have CHD and, in addition, may have a larger impact owing to the donor-recipient size discrepancy in thoracic cavity. Likewise, other factors such as pretransplant medical factors, including the number of prior cardiac surgeries and a diagnosis of CHD, were important predictors across various models and outcomes. Previous studies have shown that a single-ventricle physiology secondary to hypoplastic left heart syndrome influences outcomes; however, this was not the case in our study. In addition, longer waitlist duration likely secondary to medical or surgical factors, such as organ dysfunction, human leukocyte antigen sensitization or mismatch, and the need for other procedures were important factors in the predictive models. These medical factors have been similarly identified in prior research using ML approaches in other transplantation data, including those of adult populations [15,28,41-43]. Patient social factors predicting outcomes across the time frames in this study included age, ethnicity, level of education, and sex, which have been reported as important predictors in prior research [15,28,41-43]. Female and adolescent patients have been shown to be at greater risk for rejection episodes [44-46] and mortality than male or younger patients [47-51]. Our study also highlighted that recipient ethnicity was an important predictor for 5-year mortality. Obviously, it is difficult to predict why that is the case, but it does call for a need to further understand the complex interplay of various psychosocial factors.

### Improving Future Modeling

Our modeling efforts build on prior studies through the inclusion of posttransplant data through subsequent observation windows using TRF data. Despite this, posttransplant health outcomes for children and adolescents remain challenging to predict with better-than-modest accuracy. The UNOS data constitute a large and valuable registry of transplant patients nationally, yet this administrative database *as is* may not be optimal for prediction of specific posttransplant health outcomes owing to the lack of granularity at important clinical time points [43]. Importantly, these data sets also lack important data collected on psychological, social, and environmental factors, which can help predict long-term outcomes. In addition to medical factors, psychosocial variables and family functioning are well-known to influence outcomes [52-54]. Usually, psychosocial variables and family functioning are not well represented in these databases, limiting an important aspect of care, which affects opportunities for effective predictive modeling. Despite the importance of psychological and social determinants of posttransplant pediatric heart transplantation outcomes, these valuable data are not available in the UNOS database or in similar transplant data sets, such as the Studies of Pediatric Liver Transplantation [55] and Scientific Registry of Transplant Recipients [56] databases. The absence of such parameters can likely affect the predictive ability of these models; for example, previously, UNOS data captured physician- or transplant team–reported nonadherence (UNOS variable: *recipient noncompliant during this follow-up period* [PX_NCOMPLIANT]), but this variable has been excluded from TRF forms since 2007. Although physician proxy reports, reports, or opinion of patient medication adherence have inherent measurement issues [13], the lack of this critical predictor from these data sets and our inability to include these in modeling algorithms is a major loss in predictive utility, especially because of the known strong association between medication nonadherence and numerous posttransplant outcomes [2-5,50,57,58]. To overcome these limitations, the inclusion of granular longitudinal structured and unstructured clinical and psychosocial data within the patient EHR (eg, text from clinical notes) using these advanced analytical methods is the next step to refine the modeling algorithms, thereby increasing chances of better predictive capability.

### Limitations

This study has several limitations, including the inherent ones related to the use of database and registry data; for example, all rejections were treated as though they were of the same grade. In this work, we treated the 3 outcomes independently, although 1 outcome may in fact be a cause of another. Nonetheless, we built different models for different outcomes. In future work, we will build multiclass models with different combinations of outcomes as the prediction outcome. In this work, we grouped together patients in the UNOS database from 1987 to 2019. In future work, we will account for era and changes in clinical practice and ways to determine outcomes. This work aims to demonstrate the promise and limitations of using ML compared with using registry data in predicting posttransplantation outcomes in pediatric recipients. Because of the number of models and algorithms we evaluated, we used default parameters for the ML algorithms. With further hyperparameter tuning, we may be able to further improve the prediction performance of these models. We also converted categorical variables to numeric variables when building the prediction models. Another approach would have been to use a one-hot coding scheme for all categorical variables. However, because of the small sample size, number of categorical variables, and number of categories in these variables, one-hot coding would have resulted in a very sparse data set. Nonetheless, we created one-hot variables for 8 important diagnoses for transplantation outcome prediction.

### Conclusions

This study evaluates the approaches of 7 ML models and 1 DL model to predict posttransplant health outcomes using patient-level data and demonstrates the advantages and limitations of current methods to inform pediatric heart transplantation care. Important outcomes can be predicted with reasonable accuracy using various modeling techniques, and our study presents a comprehensive comparison of these techniques. We evaluated the approaches of these 8 models for 6 post–heart transplantation outcomes (organ rejection and mortality at 1, 3, and 5 years). Among the models for predicting these 6 outcomes, XGBoost yielded better AUPRC values than the other models in 3 of the 6 outcomes (ie, AUPRC 0.739 for 3-year rejection, AUPRC 0.888 for 5-year rejection, and AUPRC 0.575 for 5-year mortality). The NN outperformed other models in 2 outcomes (ie, AUPRC 0.868 for 1-year mortality and AUPRC 0.600 for 3-year mortality). The SVM performed slightly better than the NN in 1-year rejection prediction (AUPRC 0.614). Currently, the DL methods have not demonstrated additional predictive accuracy compared with the SVM, RF, and MLP methods. Future research should continue to seek out rich data sources such as EHRs to improve granularity and integrate them with existing registry data, using advanced analytical methods for predictive modeling of outcomes for pediatric HT recipients. Moreover, clinical notes in EHRs contain a wide range of social determinants of health for patients. We will develop a natural language processing pipeline to extract such information and enrich the prediction models for social risk stratification.

## Appendix

Multimedia Appendix 1 PRISMA (Preferred Reporting Items for Systematic Reviews and Meta-Analyses) chart for identifying the related work; description of variables; coding for categorical variables; and the rank and significance of top 20 variables having higher impact on prediction by outcomes, prediction windows, and machine learning (ML) algorithms according to Shapley additive explanations (SHAP) values.

Multimedia Appendix 2 Impact of the top 20 variables on rejection and mortality prediction by mean (|SHAP value|) for the random forest model. (A) Rejection: 1-year window. (B) Rejection: 3-year window. (C) Rejection: 5-year window. (D) Mortality: 1-year window. (E) Mortality: 3-year window. (F) Mortality: 5-year window. SHAP: Shapley additive explanations.

## Abbreviations

AdaBoost: adaptive boosting
AUPRC: area under the precision-recall curve
AUROC: area under the receiver operating characteristic curve
CHD: congenital heart defect
CV: cross-validation
DL: deep learning
EHR: electronic health record
HT: heart transplant
LAR: late acute rejection
LR: logistic regression
ML: machine learning
MLP: multilayer perceptron
NN: neural network
PRISMA: Preferred Reporting Items for Systematic Reviews and Meta-Analyses
ReLU: rectified linear unit
RF: random forest
SHAP: Shapley additive explanations
SVM: support vector machine
TRF: transplant recipient follow-up
UNOS: United Network for Organ Sharing
XGBoost: extreme gradient boosting

## Data Availability

The data underlying this paper cannot be shared publicly owing to privacy concerns because the data contain private health information of individuals receiving care from a single transplant center in the southwestern United States. However, these data are contained within the national Organ Procurement and Transplantation Network (US Department of Health and Human Services) database [59].
