# Supplementary material for: Prediction of Outcomes After Heart Transplantation in Pediatric Patients Using National Registry Data: Evaluation of Machine Learning Approaches
Source: JMIR Cardio. 2023 Jun 20;7:e45352. doi: 10.2196/45352 (PMC10334720; doi:10.2196/45352)
Supplement: Multimedia Appendix 1 [file cardio_v7i1e45352_app1.pdf]

Multimedia Appendix 1

Figure S1. PRISMA Chart for identifying the related work.

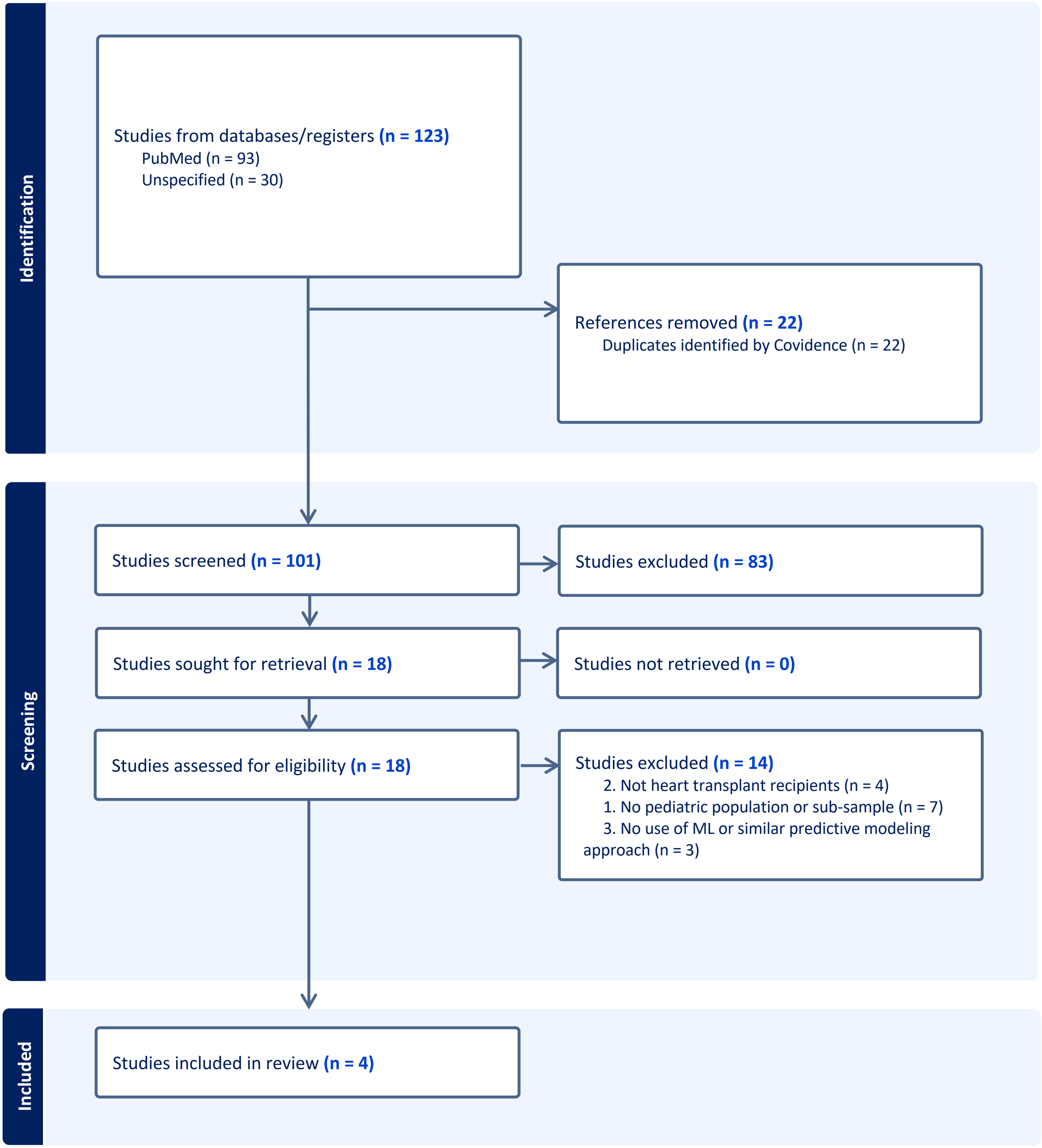

Table S1. Description of variables

Features	Type (C: Categorical; N: Numeric)	Description
Donor Variables		
abo_don	C	DONOR BLOOD TYPE
age_don	N	DONOR AGE (YRS)
bmi_don_calc	N	Donor BMI - Pre/At Donation Calculated
clin_infect_don	C	DECEASED DONOR-CLINICAL INFECTION (Y,N)
cmv_don	C	DONOR SEROLOGY ANTI CMV (FOR LIVING DONOR, PRE UNET DATA ONLY)
cod_cad_don	C	DECEASED DONOR-CAUSE OF DEATH
creat_don	N	DECEASED DONOR-TERMINAL LAB CREATININE
diabetes_don	C	DECEASED DONOR-HISTORY OF DIABETES (Y,N)
ethcat_don	C	DONOR ETHNICITY CATEGORY
gender_don	C	DONOR GENDER
hbv_sur_antigen_don	C	DONOR HEP B SURFACE ANTIGEN
hgt_cm_don_calc	N	CALCULATED DONOR HEIGHT (CM)
hist_hypertens_don	C	DECEASED DONOR-HISTORY OF HYPERTENSION
tbili_don	N	DECEASED DONOR-TERMINAL TOTAL BILIRUBIN
wgt_kg_don_calc	N	CALCULATED DONOR WEIGHT (KG)
Recipient Variables		
abo	C	RECIPIENT BLOOD GROUP @ REGISTRATION
age	N	RECIPIENT AGE (YRS)
bmi_tcr	N	BMI AT LISTING
cardarrest_neuro	C	DECEASED DONOR-CARDIAC ARREST POST BRAIN DEATH

creat_trr	N	RECIPIENT SERUM CREATININE AT TIME OF TX
days_stat1a	C	DAYS IN STATUS 1A
dayswait_chron	N	TOTAL DAYS ON WAITING LIST
diab	C	RECIPIENT DIABETES @ REGISTRATION
diag_CARDIOMYOPATHY	C	RECIPIENT PRIMARY DIAGNOSIS: CARDIOMYOPATHY
diag_CHD_WITH_HLHS	C	RECIPIENT PRIMARY DIAGNOSIS :
diag_CHD_WITH_PRIOR_SURGERY	C	RECIPIENT PRIMARY DIAGNOSIS:
diag_CONGENITAL_HEART_DEFECT	C	RECIPIENT PRIMARY DIAGNOSIS:
diag_DILATED_MYOPATHY	C	RECIPIENT PRIMARY DIAGNOSIS:
diag_HYPERTROPHIC_CARDIOMYOPATHY	C	RECIPIENT PRIMARY DIAGNOSIS:
diag_OTHER	C	RECIPIENT PRIMARY DIAGNOSIS:
diag_RESTRICTIVE_MYOPATHY	C	RECIPIENT PRIMARY DIAGNOSIS:
dial_prior_tx	C	Calculated: Ever Dialysis Prior Tx?
distance	N	DISTANCE FROM DONOR HOSP TO TX CENTER (Nautical Miles)
ecmo_tcr	C	PATIENT ON LIFE SUPPORT - ECMO @ REGISTRATION
ecmo_trr	C	PATIENT ON LIFE SUPPORT - ECMO @ TRANSPLANT
education	C	RECIPIENT HIGHEST EDUCATIONAL LEVEL @ REGISTRATION
ethcat	C	RECIPIENT ETHNICITY CATEGORY
gender	C	RECIPIENT GENDER
grf_stat	C	GRAFT STATUS
hbv_sur_antigen	C	RECIPIENT HEP B SURFACE ANTIGEN
hcv_serostatus	C	RECIPIENT HEP C STATUS
hemo_pa_mn_trr	N	MOST RECENT HEMODYNAMICS PA

		(MEAN) MM/HG @ TRANSPLANT
hgt_cm_calc	N	CALCULATED RECIPIENT HEIGHT(cm)
iabp_tcr	C	PATIENT ON LIFE SUPPORT - IABP @ REGISTRATION
iabp_trr	C	PATIENT ON LIFE SUPPORT - IABP @ TRANSPLANT
infect_iv_drug_trr	C	INFECTION REQUIRING IV DRUG THERAPY (WITHIN 2 WEEKS PRIOR TO TRANSPLANT)
inotrop_vaso_mn_trr	C	TRR MEAN PULMONARY ARTERY MEASUREMENT OBTAINED WHILE ON INOTROPES OR VASODILATERS Y/N
inotrop_vaso_pcw_tcr	C	MOST RECENT PCW (MEAN) MM/HG INOTROPES/VASODILA TORS YES/NO AT LISTING
inotropes_tcr	C	IV INOTROPES @ REGISTRATION
inotropes_trr	C	IV INOTROPES @ TRANSPLANT
ischtime	N	ISCHEMIC TIME IN HOURS
malig_trr	C	RECIPIENT ANY KNOWN MALIGNANCIES SINCE LISTING @ TRANSPLANT
most_rcnt_creat	N	PATIENT MOST RECENT ABSOLUTE CREATININE AT LISTING
prior_card_surg_tcr	C	TCR PRIOR CARDIAC SURGERY AT LISTING (NON-TRANSPLANT)
prior_card_surg_trr	C	TRR CARDIAC SURGERY BETWEEN LISTING AND TRANSPLANT (NON-TRANSPLANT)

steroid	C	CHRONIC STEROID USE Y/N/U @ TRANSPLANT
tbili	N	MOST RECENT SERUM TOTAL BILIRUBIN @ TRANSPLANT
tot_serum_album	N	PATIENT TOTAL SERUM ALBUMIN @ REGISTRATION (pre 1/1/2007 for adult)
transfusions	C	EVENTS OCCURRING BETWEEN LISTING AND TRANSPLANT: TRANSFUSIONS Y/N/U
vad_device_ty_tcr	C	CANDIDATE TYPE OF VAD DEVICE AT LISTING
vad_device_ty_trr	C	TRR VAD DEVICE TYPE
vent_support_trr	C	TRR EPISODE OF VENTILATORY SUPPORT
ventilator_tcr	C	PATIENT ON LIFE SUPPORT - VENTILATOR @ REGISTRATION
ventilator_trr	C	PATIENT ON LIFE SUPPORT - VENTILATOR @ TRANSPLANT
wgt_kg_calc	N	CALCULATED RECIPIENT WEIGHT (kg)
Donor-Recipient Variables		
abo_mat	C	DONOR-RECIPIENT ABO MATCH LEVEL
hgt_ratio_rep2don	N	Ratio of height: recipient to donor
hlamis	C	HLA MISMATCH LEVEL
wgt_ratio_rep2don	N	Ratio of weight: recipient to donor

Table S2. Coding for categorical variables (Seem to miss some categorical variables)

Variables	Coding
Donor Variables	
abo_don	
clin_infect_don	1="N" 2="U" 3="Y"
cmv_don	1="I" 2="N" 3="ND" 4="P" 5="U"
cod_cad_don	1="ANOXIA" 2="CEREBROVASCULAR/STROKE" 3="HEAD TRAUMA" 4="CNS TUMOR" 5="OTHER SPECIFY"
diabetes_don	1="N" 2="U" 3="Y"
ethcat_don	1="White" 2="Black" 4="Hispanic" 5="Asian" 6="Amer Ind/Alaska Native" 7="Native Hawaiian/other Pacific Islander" 9="Multiracial" 998="Unknown"
gender_don	1="F" 2="M"
hbv_sur_antigen_don	1="C" 2="N" 3="ND" 4="P" 5="U"
hist_hypertens_don	1="N" 2="U" 3="Y"
Recipient Variables	
abo	
cardarrest_neuro	1="N" 2="U" 3="Y"
diab	1="No" 2="Type I" 3="Type II" 4=" Type Unknown" 5="Diabetes Status Unknown"
diag_CARDIOMYOPATHY	1="Y" 0="N"
diag_CHD_WITH_HLHS	1="Y" 0="N"
diag_CHD_WITH_PRIOR_SURGERY	1="Y" 0="N"
diag_CONGENITAL_HEART_DEFECT	1="Y" 0="N"
diag_DILATED_MYOPATHY	1="Y" 0="N"
diag_HYPERTROPHIC_CARDIOMYOPATHY	1="Y" 0="N"
diag_OTHER	1="Y" 0="N"
diag_RESTRICTIVE_MYOPATHY	1="Y" 0="N"
dial_prior_tx	1="N" 2="U" 3="Y"
ecmo_tcr	1="Y" 0="N"
ecmo_trr	1="Y" 0="N"
education	1="NONE" 2="GRADE SCHOOL (0-8)" 3="HIGH SCHOOL (9-12) or GED" 4="ATTENDED COLLEGE/TECHNICAL SCHOOL" 5="ASSOCIATE/BACHELOR DEGREE" 6="N/A (< 5 YRS OLD)" 7="UNKNOWN"
ethcat	1="White" 2="Black" 4="Hispanic" 5="Asian" 6="Amer Ind/Alaska Native" 7="Native Hawaiian/other Pacific Islander" 9="Multiracial" 998="Unknown"
gender	F="Female" M="Male"
grf_stat	1="N" 2="Y"
hbv_sur_antigen	1="N" 2="ND" 3="P" 4="U"
hcv_serostatus	1="N" 2="ND" 3="P" 4="U"
iabp_tcr	1="Y" 0="N"

iabp_trr	1="Y" 0="N"
infect_iv_drug_trr	1="N" 2="U" 3="Y"
inotrop_vaso_mn_trr	1="N" 2="Y"
inotrop_vaso_pcw_tcr	1="N" 2="Y"
inotropes_tcr	1="Y" 0="N"
inotropes_trr	1="Y" 0="N"
malig_trr	1="N" 2="U" 3="Y"
prior_card_surg_tcr	1="N" 2="U" 3="Y"
prior_card_surg_trr	1="N" 2="U" 3="Y"
steroid	1="N" 2="U" 3="Y"
transfusions	1="N" 2="U" 3="Y"
vad_device_ty_tcr	1="NONE" 2="LVAD" 3="RVAD" 4="TAH" 5="LVAD+RVAD" 6="LVAD/RVAD/TAH Unspecified"
vad_device_ty_trr	1="NONE" 2="LVAD" 3="RVAD" 4="TAH" 5="LVAD+RVAD" 6="LVAD/RVAD/TAH Unspecified"
vent_support_trr	1="N" 2="U" 3="Y"
ventilator_tcr	1="Y" 0="N"
ventilator_trr	1="Y" 0="N"
Donor-Recipient Variables	
abo_mat	1="Identical" 2="Compatible" 3="Incompatible"
hlamis	1=0 2=1 3=2 4=3 5=4 6=5 7=6

Table S3. Rank and significance of top 20 variables having higher impact on prediction by outcomes, prediction windows and ML algorithms according to SHAP values

Variables	Group	Rejection			Mortality			# of Models	Avg. Rank
		1-Yr	3-Yr	5-Yr	1-Yr	3-Yr	5-Yr		
grf_stat	Recipient	1***	1***	2***	1***	1***	1***	6	1.2
days_stat1a	Recipient	3**	2***	3***	4	2***	2***	6	2.7
vad_device_ty_trr	Recipient	2***	4***	7***	3***	4***	4***	6	4.0
malig_trr	Recipient	10***	5***	5***	2***	3***	3***	6	4.7
tbili	Recipient	11**	7**	18*	9***	8***	11***	6	10.7
hgt_cm_calc	Recipient	5***	15***	14**	8***	7**	16	6	10.8
ischtime	Recipient	18	13	6**	16	16	18	6	14.5
distance	Recipient	7	20*	20	20	13	8**	6	14.7
prior_card_surg_trr	Recipient	4***	3***	1***		5***	5***	5	3.6
cardarrest_neuro	Recipient	16***	9***		10***	11***	7***	5	10.6
bmi_tcr	Recipient	13**	12***		6	14	9**	5	10.8
hgt_ratio_rep2don	Recipient-to-Donor		14	11	5***	10**	17	5	11.4
creat_trr	Recipient	14**	10***	15*		12***	10*	5	12.2
wgt_kg_calc	Recipient	8***	16***		11***	15	14	5	12.8
wgt_kg_don_calc	Donor	15***	19***	12*	7***	18		5	14.2
dayswait_chron	Recipient		11	4	18	17		4	12.5
hgt_cm_don_calc	Donor	12***	8***	16***	14***			4	12.5
wgt_ratio_rep2don	Recipient-to-Donor	19	17*	10		19		4	16.3
bmi_don_calc	Donor	20		17	15***		19	4	17.8
prior_card_surg_tcr	Recipient		6***	9***			6***	3	7.0
age_don	Donor	6***				9	13***	3	9.3
tot_serum_album	Recipient	17	18	8				3	14.3
clin_infect_don	Donor					6***	12***	2	9.0
tbili_don	Donor			13	17***			2	15.0
hemo_pa_mn_trr	Recipient			19	12			2	15.5
diag_CONGENITAL_HEART_DEFECT	Recipient				13***	20***		2	16.5
age	Recipient	9***						1	9.0
most_rcnt_creat	Recipient						15	1	15.0
creat_don	Donor				19			1	19.0
ethcat	Recipient						20***	1	20.0

Note: *** at significance level of 0.01; ** at significance level of 0.05; * at significance level of 0.1. # of Models means number of models including the corresponding variable in top 20 variables in terms of mean (|SHAP value|).
