# Supplementary figures and images for: Prediction of Outcomes After Heart Transplantation in Pediatric Patients Using National Registry Data: Evaluation of Machine Learning Approaches
Source: JMIR Cardio. 2023 Jun 20;7:e45352. doi: 10.2196/45352 (PMC10334720; doi:10.2196/45352)

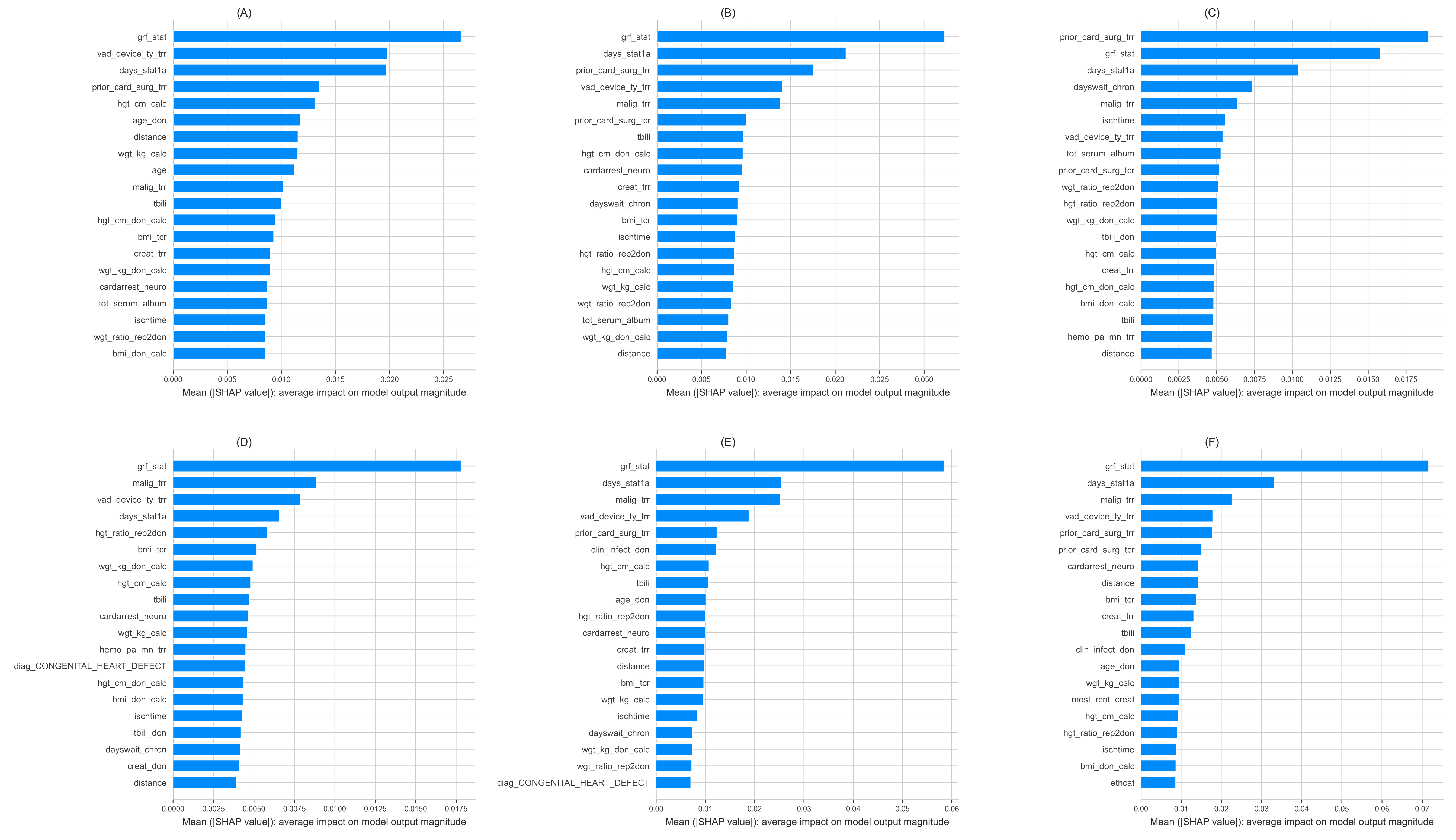

Supplement: Multimedia Appendix 2 [file cardio_v7i1e45352_app2.png]
